# Perspectives of traditional Chinese medicine to patch up immune checkpoint blockers

**DOI:** 10.37349/etat.2022.00107

**Published:** 2022-10-31

**Authors:** Shiu Ying Tsao

**Affiliations:** Department of Clinical Research, Hong Kong SAR Oncology Centre, Hong Kong SAR 999077, China; Tianjin Medical University General Hospital, China

**Keywords:** Antiangiogenesis, hypoxia-inducible factors, multi-agent approach, traditional Chinese medicine, tumor microenvironment, vascular endothelial growth factor

## Abstract

In this era of cancer immunotherapy, the response rates of immune checkpoint blockers (ICBs) are still too low and the adverse events may also be significant. Of the ways of patching up such deficits, chemotherapy (ChT), especially if metronomic, seems promising, especially as immunity induced by immunogenic cell death (ICD) may be preserved. However, side effects, e.g., lymphocytopenia and interstitial pneumonitis cannot be ignored; eventually, resistance may also ensue. Vascular endothelial growth factors (VEGFs), being potent angiogenic factors, promote cancer cells’ purposeful angiogenesis rendering an extremely resistant tumor microenvironment (TME). This highly evasive and extremely resilient TME actually demands multi-agent, multi-target agents as currently in use through traditional Chinese medicine (TCM). With a good track record of 3,000 years, TCM is favored by mainland Chinese cancer patients. Although TCM had been criticized as unscientific and imprecise, recently, artificial intelligence (AI) technologies serve to elucidate the sound scientific basis and validity of TCM. Several TCM preparations having anti-VEGF actions are found; others suppress immune checkpoints. Especially, these herbs’ multi-prong approach appears to be more effective than Western medicine’s primarily monotherapy approach if one wishes to eradicate the very resistant TME. A “bonus” point is that some autoimmune-related adverse side effects of ICBs may also be reduced by TCM. Nevertheless, as the TCM experience is mostly anecdotal, robust clinical trials are mandatory. Moreover, other TCM problems, e.g., herbal batch variations and consistency and uniformity of herbal prescriptions are outstanding. Invariably, TCM prescriptions have daily variations as the practice of “syndrome differentiation” is hailed. Despite experienced TCM practitioners would refuse to give up their time-honored traditional practice, the multi-prong approach is still very attractive for the undue resilience of TME, let alone its good safety profile, ready availability, and eminent affordability. Although the passage is dark, light is now appearing at the end of the tunnel.

## Introduction

The management of cancer has now entered a new era in which immunotherapy, mainly using immune checkpoint blockers (ICBs), has clearly taken the center stage. Yet, well-known deficiencies still exist. Apart from considerable adverse events, the low response rates constitute a greater concern. For instance, the objective response rate (ORR) of pembrolizumab (an ICB) for advanced cervical cancer was only 12.2% [1]. For advanced endometrial cancer, a more recent clinical trial on dostarlimab showed a more impressive ORR of 43.5% for cases with mismatch repair deficient/microsatellite/instability-high (dMMR/MSI-H) but the ORR was still only 14.1% for those with mismatch repair proficient/stable (MMRp/MSS) [2]. Although dMMR/MSI-H may effectively indicate adequate tumor-infiltrating lymphocytes (TILs) (see below), only up to 30% of endometrial carcinoma patients are actually in this favorable category [3]. Clearly, the vast majority of these cancer patients would not benefit from ICBs. Such low response rates would effectively deprive most cancer patients of the benefit of ICBs. Especially, any cancer patients with pre-existing autoimmune disorders would effectively constitute a contradiction to ICBs. This may thus further reduce the usefulness of ICBs. Recently, it is progressively recognized that one of the most important culprits causing undue resistance to cancer therapy agents is the tumor microenvironment (TME). It is so evasive that even monotherapy agents targeting specific TME parameters are hardly successful [4]. As overcoming the TME is now deemed to be highly relevant, a better understanding of it is most appropriate. The valid points are highlighted here while attempting to reduce resistance to ICBs. To begin with, remedies like antiangiogenic agents to normalize makeshift tumor vasculatures of the TME would be covered. Meanwhile, in combination with chemotherapy (ChT), especially metronomic ChT (mChT), anti-vascular endothelial growth factors (anti-VEGFs), e.g., bevacizumab, antiangiogenic agents of some tyrosine kinase inhibitors (TKIs), e.g., sorafenib [5], as well as gut microbiota would be discussed. Especially, the controversial but possibly highly relevant role of traditional Chinese medicine (TCM) would be discussed in some detail. Admittedly, apart from attempting to overcome the TME, it is also prudent to avoid adding further toxicity to existing adverse events of ICBs. Lastly, with the undue mystery and considerable complexity of TCM, a lengthy (but boring and hardly comprehensible) account covering the very vague practice and nature of TCM is purposely avoided. Rather, TCM is being introduced at various relevant points but eventually, an appropriate conclusion is reached.

## The “self-centered” tumor

### The makeshift angiogenesis

As cancer cells would initially begin with minimal numbers, they would usually survive on shared oxygen and nutrients from the host’s existing blood vessels. However, as the number of cancer cells gradually increases, the cumulative larger number of cancer cells would naturally require an induction of new blood vessels so as to obtain their own share of oxygen and nutrients. Actually, the induced blood vessels are usually makeshift in nature and mostly too porous or leaky due to poor quality pericytes [6, 7]. Intriguingly, cancer cells can exploit such makeshift blood vessels to their own advantage. Actually, cancer cells can readily thrive in such very inadequate environments even better than normal cells. Moreover, such poor environments would render a hypoxic and acidic surrounding which also has excessive lactates due to anaerobic glycolysis (Table 1). This very hostile environment would even serve to suppress host immunity. In any case, with the leaky vessels unable to deliver adequate host immune cells, the cancer cells can thus thrive happily. Taken together, the tumor environment would preclude most normal functions of host cells. Moreover, as the so-called “aerobic glycolysis” means cancer cells can readily switch to the glycolysis cycle even in the presence of oxygen (unlike normal cells that would only have anaerobic glycolysis cycles) it would actually be futile to “starve” cancer cells by glucose deprivation, especially as the TME actually has many “plan Bs” and “plan Cs”, and ample viable alternative pathways in case one particular pathway fails. Apparently, only a multi-target approach could overcome the very evasive TME more effectively.

**Table 1.** The main sequence of events occurring in the TME

**No.**	**Parameter**	**Initial result**	**Subsequent result**	**Reference**
1	Angiogenesis	Leaky vessels	Hypoxia	[6]
2	Hypoxia	Induce HIF	Increased angiogenesis	[8]
3	Insufficient glucose	Anaerobic glycolysis	Lactate increase	[9]
4	Excessive lactate	Increased acidity	Increased influx of MDSCs, TAMs, Tregs^*^	[10]
5	Anaerobic glycolysis	Lactate increase	Acidity increase	[7]
6	Poor pericytes	Leaky vessels	Increased interstitial fluid pressure	[7]

Items 1 and 2 would readily form a vicious cycle of angiogenesis, hypoxia, worse angiogenesis, worse hypoxia, etc. * together, these cells would significantly reduce host immunity; HIF: hypoxia-inducible factor; MDSCs: myeloid-derived suppressor cells; TAMs: tumor-associated macrophages; Tregs: regulatory T cells

### The TME: deemed to be the worst culprit for cancer resistance

**Figure 1.**
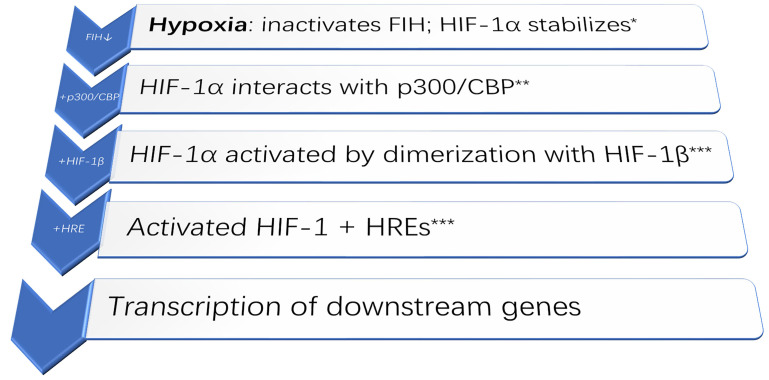
Schematic diagram of the main molecular event upon hypoxia. HIF-1α + HIF-1β + HREs would activate the transcription of downstream genes [12]. * FIH: factor inhibiting HIF [13]; ** p300/CBP: p300/CREB binding protein (transcriptional coactivators) [14]; *** HREs: hypoxia-response elements; “↓”: inhibited; “+”: added on

**Figure 2.**
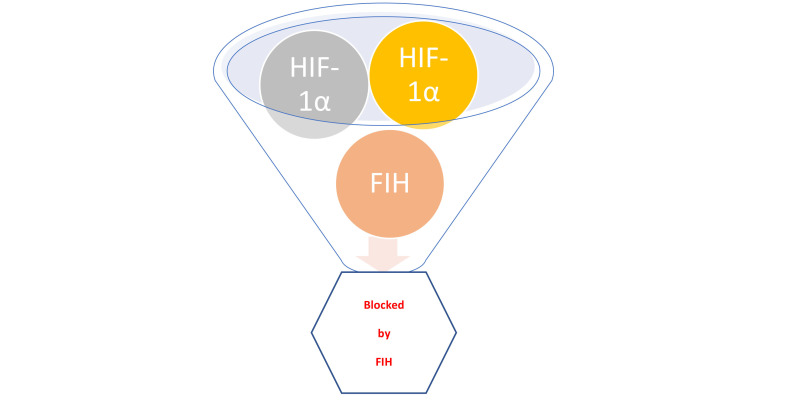
HIF-1α blockade. In normoxic environments, HIF-1α is blocked by FIH [15]

**Figure 3.**
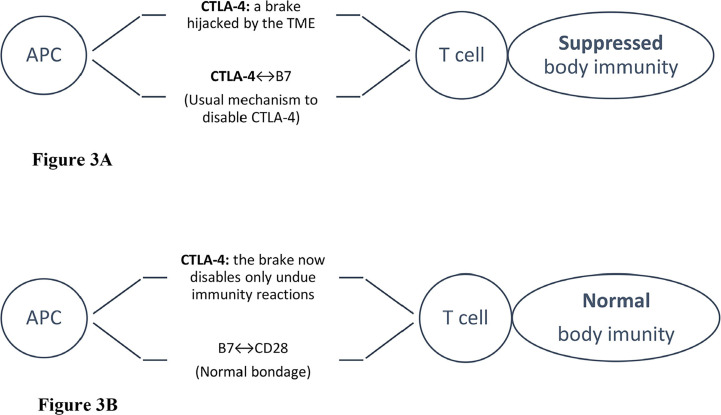
Scheme of CTLA-4 blockade. Tumor cells “hijack” immune checkpoints to disable host immunity. Illustrated are the schemes of an APC attempting to present tumor-associated antigens to T cells. However, with normal immunity, the CD28 co-activating receptors on T cells would be bound to B7 proteins on APCs except for the checkpoint braking effect, e.g., by CTLA-4. When CTLA-4 binds to B7, the host immunity effect is disabled. Upon administering an anti-CTLA-4 (an ICB), the effect would be reversed, i.e., no more braking effect as B7 is now bound to CD-28 again, clearly to the tumor cells’ great disappointment [16]. APC: antigen-presenting cell; CTLA-4: cytotoxic T-lymphocyte-associated-4; “↔”: binding to

Despite the well-established necessity for oxygen, how living cells actually sense hypoxia has only been discovered in the late 1990s, with the 2019 Nobel Prize in Physiology or Medicine going to William G. Kaelin Jr., Sir Peter J. Ratcliffe, and Gregg L. Semenza [11]. Their discoveries centered on the HIF: a vital transcription factor that regulates gene expression while responding to hypoxia. Notably, hypoxia induces angiogenesis through various HIF mechanisms. Importantly, in response to hypoxia, metabolic adaptations of cancer cells by HIF-1, e.g., switching from oxidative phosphorylation to glycolysis and also metabolizing glutamine (in preference to glucose) for lipid synthesis, a drop in extracellular pH would then occur. All of these are actually involved in enhancing tumor progression and even during metastasis. Notably, HIF-1 also plays a vital role in the immune response: its induction is crucial for the infiltration and activation of myeloid cells. Moreover, HIF-1α regulates the rather delicate balance between Tregs and T helper 17 (Th17) (pro-inflammatory) cells. Rather similar to cancer cells which adapt their metabolism to hypoxia, the HIF-1α-dependent metabolic switch to glycolysis may promote production of Th17 cells but would effectively suppress Treg generation. The various regulatory pathways involved during hypoxia are reviewed by Ziello and colleagues [8]; a schematic representation of molecular events is in Figure 1. Admittedly, as these related molecular events are rather complex, further schematic representations to illustrate the most salient features are in Figures 2 and 3.

The TME is now recognized to be an extremely resilient, smartly evasive and highly resistant “haven” for cancer cells. Without adequate TILs, these tumors are actually too “cold” to have any adequate response to ICBs: the main reason why ICBs fail [17, 18]. As leaky vessels also lead to high interstitial fluid pressures, the TME is thus characterized by hypoxia, acidity, and high interstitial fluid pressures. Remarkably, low-dose anti-VEGF agents, e.g., bevacizumab have been found to render vessel normalization for the makeshift tumor vasculature. This may effectively overcome hypoxia and increase the bioavailability of drugs, let alone facilitating the trafficking of antitumor immune cells [19–22]. Actually, these antiangiogenic agents have also been tried clinically before the debut of ICBs. Despite the makeshift vasculature giving rise to hypoxia, acidity, and high interstitial fluid pressures [7], hopefully, a skillful administration of these antiangiogenic agents would not only render the unique effect of normalizing the vasculature but also boost the response rates of ICBs [17]. One of the most effective anti-VEGFs, e.g., bevacizumab, is a potent antiangiogenic agent (see below). The function of anti-VEGF agents is not just to inhibit the formation of new blood vessels but also to function even more appropriately to normalize disorderly blood vessels [19, 23].

Interestingly, the inadequacy of pericytes rendering makeshift tumor vessels too porous for effective perfusion is actually similar to that of cerebral edema, as induced by “secretive meningiomas” [24, 25]. Although the detailed pathophysiology of cerebral edema remains to be elucidated [24], evidently, leaky vessels would occur within the brain, especially after head injury. Apparently, secretory meningiomas patients would have their blood vessel pericytes indirectly affected and cerebral edema occurred well beyond the meningiomas. On microscopy, these meningiomas were found to have abnormal pericytes [25]. Apparently, the peculiar pathophysiology of these secretory meningiomas was closely related to pericytes and the subsequent diffused, “oversized” cerebral edema was actually out of proportion to the actual much smaller size of those meningiomas.

Moreover, as poor perfusion leads to hypoxia and, in turn, attracts cells like MDSCs, also known as the “queen bee” of the TME [10] as well as other related cells, e.g., TAMs and Tregs. Unfortunately, these cells in unduly hypoxic environments may also secrete growth factors and anti-inflammatory cytokines that have profound immunosuppressive effects including increased expression of programmed death-1 (PD-1) receptors, let alone the induced HIFs would promote PD-1 receptors [26, 27]. Ultimately, cancer-associated fibroblasts (CAFs) develop: they not only enhance tumor invasion and dissemination but also promote cancer cell proliferation, let alone contributing actively to tumor resistance.

## ICB’s low response rates

### Enhancement by ChT or antiangiogenic agents

Although theoretically, ICBs are designed to inhibit specific immune checkpoints, clinically, the response rates are too low to benefit most cancer patients, i.e., tumors are too “cold” to respond, as alluded to above. Occasionally, chemotherapeutics or antiangiogenic agents are combined with ICBs to boost response rates. However, there are considerable limitations, as have already been described by Tsao [28]. Briefly, for TKIs, there may be a problem with adverse events upon combining, e.g., lymphocytopenia and excessive interstitial pneumonitis that had rendered some combination trial suspensions; eventually, resistance may also ensue. Moreover, the usual dose of ChT may be too high and could even suppress immunity rendered by ICBs. Appropriately, for the combination, mChT appears more appropriate. Although high-dose antibiotics are often successful, e.g., for serious bacterial infections, maximum tolerated dose (MTD) cancer ChT can hardly achieve equivalent success. Currently, this discrepancy is recognized to be due to the notoriously resistant TME. Actually, ChT may also induce immunogenic cell death (ICD) [29] and may even enhance ICBs, especially mChT (less suppressive effect on induced immunity) is employed.

On the other hand, although antiangiogenic agents, especially anti-VEGF ones, are originally designed to prune the tumor’s neo-vasculature, they have recently been found to render normalization of the makeshift tumor vasculature by pruning mainly abnormal vasculature but leaving the good ones behind. The proviso is that the low-dosage is used. Although the labeled dosage of, e.g., bevacizumab (15 mg/kg) works best for pruning neo-vasculature, for vessel normalization, a low-dosage is much more appropriate [6, 19, 23]. This is also in line with personal and other published experiences (see below). Desirably, the best blood vessel normalization is to render a “cold” tumor “hot” through a better influx of TILs to it.

### Possible enhancement by low-dose bevacizumab

Although the pathophysiology of cerebral edema is complex (as alluded to), apparently, pericyte abnormality is indirectly associated with leaky blood vessels in cerebral edema. In clinical practice, early cerebral edema would usually respond to steroids or osmotic agents (e.g., mannitol). However, for resistant cases, although novel agents have now been proposed, clinically, the personal anecdotal experience incidentally tallies with published ones that low-dose bevacizumab usually works surprisingly well even for those resistant cerebral edema cases [30–32]. Admittedly, off-label drug prescription is to be discouraged, except the prompt and effective relief of these resistant cerebral edema cases by low-dose bevacizumab appears monumental. However, robust clinical trials on such a particular effect are difficult, due to the rarity of these cases. Remarkably, such anecdotal clinical experience may serve to verify that low-dose bevacizumab may well be highly effective for leaky blood vessels that are characteristic of cerebral edema [24]. Realistically, the labeled dose of bevacizumab (15 mg/kg) is for pruning neo-vasculature but the off-label dose (7.5 mg/kg) is for normalizing vasculature to reduce the interstitial fluid pressure so useful for managing the TME.

### The price of enhancement

Despite the “bonus” effect of off-label anti-VEGF agents, i.e., bevacizumab that may render normalization of makeshift vasculature, the survival rates achieved by these monotherapy anti-VEGF agents have not been impressive [33]. This is likely to be due to the TME’s very remarkable evasiveness and ample alternative escape pathways. Although combining anti-VEGF agents with ICBs would enhance the response rates, unacceptable side effects usually ensue, as reviewed by Tsao [28]. Notably, even though more novel TKIs, e.g., sorafenib also have antiangiogenic effects, they are usually less potent than anti-VEGF agents.

Recently, for hepatocellular cancer (HCC, an aggressive cancer), an anti-VEGF agent (bevacizumab 15 mg/kg) was added to atezolizumab (an ICB) to achieve higher response rates and longer survivals [34] in the IMbrave150 Trial for unresectable HCCs. However, compared with sorafenib, despite achieving a superior median overall survival (longer by 5.8 months) with the combination than monotherapy sorafenib [35], adverse effects were also considerable, as the bleeding had occurred in as many as 1/4 of patients upon combination. Retrospectively, as high-dose bevacizumab is associated with a higher bleeding rate, low-dose bevacizumab might be a better alternative.

For advanced renal cell carcinoma (RCC) patients, nivolumab (an ICB) plus cabozantinib (a multi-kinase inhibitor) is already approved for first-line treatment of RCC patients, based on the randomized CheckMate 9ER Trial for treatment-naïve but advanced RCC patients [36]. Actually, despite a better response rate of around 50% (0% for the chromophobe sub-type), some of the most common adverse events of the combination arm were palmar-plantar erythrodysesthesia (redness, swelling, and blistering of the hands and feet; 40%), hypothyroidism (34%) [37]. Unfortunately, there is a significant price to pay for these better response rates.

### Microbiota posing as “non-toxic enhancer”

Recently, it has been advocated that, perhaps combination of ICBs with gut microbiota agents may enhance ICBs and have much less adverse effects. Although pre-clinical studies of oral Bifidobacterium have been found to have significant cancer immunotherapy effects [38, 39], robust clinical trial results are required to confirm the results. Yet, the field of onco-microbial science is only just commencing [40]. Notably, fecal microbiota transplantation (FMT) is deemed as much more effective for immune enhancement and even considered as the “last resort” remedy, e.g., for resistant *Clostridium difficile* infections [41]. However, the Food and Drug Administration (FDA) has not approved FMT for any indication, even though it has been advocated for patching up ICB’s deficiencies (both low response rates and immune-related adverse events (irAEs). Apparently, FMT remains to be better developed as its immune-mediated mechanisms have yet to be well established [42]. Lastly but not the least, during this pandemic, the transmission of various infections (especially viral ones) through feces raises the most concern [43]. Notably, severe acute respiratory syndrome coronavirus 2 (SARS-CoV-2) shedding may persist despite both clinical manifestations and nasopharyngeal swabs are negative. Thus, the risks of infections through FMT are actually real especially as clearly, some stool banks screen less carefully than others for diseases possibly transmitted by FMT [44].

## TCM is distinct

### Possibility of reducing IREAs of ICBs

For adverse events of ICBs, especially irAEs, various agents are tried, most commonly steroids [45]. Disease-modifying anti-rheumatic drugs (DMARDs) might occasionally be considered but due care and concern should actually be taken and preferably, the use of DMARDs for ICB’s irAEs should actually be a joint consultative effort with a rheumatologist [46]. Although most irEAs of ICBs may be mild, some are definitely life-threatening, e.g., interstitial pneumonitis and these would usually demand immediate ICB discontinuation. Despite the availability of all these management tactics for irAEs, ICBs may have to be suspended upon the failure of all these management tactics.

Interestingly, in mainland China, TCM herbs are now actively being researched for the potential of reducing irAEs of ICBs, e.g., total glucosides of paeony and *Tripterygium wilfordii* glycosides (TWG) from *Tripterygium wilfordii* Hook F (TwHF) [47, 48]. As China’s State FDA (SFDA) has already approved total glucosides of paeony for rheumatoid arthritis [49] and TWG for Crohn’s disease, rheumatoid arthritis, and ulcerative colitis [50], these herbs are being actively studied for managing irAEs of ICBs. Notably, an important advantage of these herbs is the wide therapeutic index of TCM herbs, so the administration of these herbs for managing ICB’s irAEs may well be simpler than DMARDs. Anyway, it is already a well-known fact that in mainland China, using TCM to reduce the side effects of Western medications is rather commonplace.

### China’s two parallel medical systems benefit oncology

In mainland China, the state strategy is for both Western medicine and TCM streams to run in parallel to each other [51]. For oncology services, as medical linear accelerators (linacs) are usually considered essential for delivering modern radiotherapy, looking at the provision of linacs may give an idea on the adequacy of provision of oncology equipment. Currently, with a population of 1.4 billion, China may appear to have a suboptimal number (2,000) of linacs. Admittedly, this number is below the World Health Organization (WHO)’s requirement recommendation of 2-3 linacs/million population [52]. Moreover, China’s 1.42 linacs/million population actually compare very unfavorably with that of USA (11.4 linacs/million population). Most surprisingly, people in mainland China are hardly complaining about the inadequacy of linacs. Apparently, the provision of two parallel medical systems works well. Notably, both available systems are providing satisfactory and roughly equivalent oncology services. Thus, in mainland China, TCM is not only well respected but also has a good track record. Even in Hong Kong where the Government still maintains a priority on Western medicine, TCM is still considered as a good “second best”. Admittedly, TCM has an extremely long history of 3,000 years and is still trusted in mainland China. Finally, in perspective, the definition of TCM actually also comprises of some other modalities, e.g., acupuncture, moxibustion, acupressure, herbal poultice, “gua sha” massage (scraping to produce light petechiae), and cupping [53].

### Contribution to oncology

Although TCM especially for oncology has been criticized as “unscientific” or “imprecise”, during recent decades, more and more scientific reports validating the scientific basis of TCM have been published.

Remarkably, in 1997, China’s SFDA had already approved the use of Kanglaite (extracted from the Chinese herb, Coicis semen yokuinin) for HCCs and non-small cell lung cancers (NSCLCs) resistant to ChT. Intriguingly, this was long before the debut of anti-epidermal growth factor receptors (anti-EGFRs) in the early 2000s. It was unfortunate that anti-EGFRs had an adverse event of a hitherto unrecognized but rapidly fatal pneumonitis coined as interstitial lung disease (ILD). In Japan, with heavy use of anti-EGFRs for NSCLCs, it had even caused a significant social problem [54–56]. Retrospectively, with the good acceptance of Kanglaite in China, it is most unlikely to be an anti-EGFR, as no similarly serious side effects had been documented despite the very heavy use of Kanglaite in China. In a meta-analysis of 26 studies, 22 could be pooled and the conclusion was, combining it with ChT would increase the response rates. It was only recently that, with the recent concept of TME being better recognized, Kanglaite was eventually found to inhibit HIF-1α [57]. Admittedly, in the late 1990s, the exact mechanism of Kanglaite was hardly known at all, especially as the recent concept of TME was, at most, in its infancy by then. Nevertheless, as at that time, hardly any agents could ever enhance ChT response rates effectively, especially for cases resistant to multiple lines of treatment, let alone the belated availability of anti-EGFRs (in the early 2000s).

Another TCM preparation deserving attention is Yangzheng Xiaoji (YZXJ) capsules that comprise of 16 TCM herbs [56]. Despite a strong antiangiogenic element and even a direct effect on cancer cells, YZXJ is reported to have a good safety profile, let alone its convenient oral administration [56]. In mainland China, with an excellent albeit anecdotal efficacy for various cancers, it is well respected and hailed as an important cancer drug. Admittedly, further high-quality clinical studies are mandatory for further investigations.

Currently, TCM’s practice has usually been relying very heavily on highly complex but anecdotal combinations of numerous herbs, each having countless alkaloids [58]. Yet, despite such unique and undue complexity, not only there is reputable anecdotal efficacy throughout the centuries but also a good and time-honored safety profile. The secret probably lies in the prescription of herbs at their lowest effective dosage and, perhaps, depending on numerous mutual enhancement mechanisms, as together, they would achieve the desired effect which can be validated and may occasionally be better than Western medicine. Historically and on a related condition (infantile eczema, likely having a significant immunity basis), there was a well-organized clinical trial complete with herbal batch control and strict uniformity of herbal prescription. Surprisingly, TCM herbal concoctions were found to be significantly more effective than steroids for UK children tested [59]. Amazingly, this clinical trial had successfully implemented herbal batch variation control and even adopted uniformity of prescription which was clearly against the usual TCM practice.

### The anti-VEGF action

Recently, several TCM herbs have been discovered to have an action against VEGFs [18, 23]. Although most of these may conveniently be taken orally and the safety profile is also good, let alone the eminent affordability, a considerable research effort is clearly still required. A brief summary of some of the beneficial effects of these agents which have mainly anti-VEGF effects is in Table 2. Recently, it has also been found that some TCMs may also inhibit immune checkpoints, especially for PD-1 and PD-ligand 1 (PD-L1). A selection of these TCMs is in Table 3. Intriguingly, despite TCM’s heavy reliance on anecdotal experience throughout the ages, it has somehow achieved initial success in overcoming the notoriously resistant TME. As far as adverse effects are concerned, interestingly, unlike the very recent IMbrave150 trial, TCM does not appear to have unacceptable bleeding episodes [35]. Of course, much more investigative efforts, especially robust clinical trials are mandatory.

**Table 2.** Selected TCMs with anti-VEGF, anti-HIF, and other actions

**Compound/preparation**	**TCM herb**	**Mode of action**	***In vitro***	***In vivo***	**Cancer**	**Year**	**Ref.**
Ginsenoside	Panax ginseng	Anti-VEGF	+	+	Breast cancer	2017	[60]
Tanshinone IIA	Salvia miltiorrhiza	Anti-VEGF	+		NSCLC	2015	[14]
Curcumin	Curcuma longa	Anti-VEGF		+	Glioma	2017	[61]
Balcalein	Scutellaria baicalensis	Anti-VEGF	+	+	NSCLC	2016	[62]
Formononectin	Astragalus membranaceus	Anti-VEGF		+	Breast cancer	2015	[13]
Codonolactone	Atractylodes lancea	Anti-VEGF	+	+	Breast cancer	2015	[12]
Timosaponin AIII	Anemarrhena asphodeloides	Anti-VEGF	+	+	Various	2020	[63]
Pien Tze Huang	Mixture of TCM herbs	Anti-VEGF	+		CRC	2016	[64]
Ginsenoside	Panax ginseng	Anti-HIF^*^	+	+	Ovarian cancer	2014	[65]
Pien Tze Huang	Mixture of TCM herbs	Anti-HIF^*^	+		Colon	2014	[66]
Bufalin	Chensu	Anti-HIF^*^		+	HCC	2016	[67]
Kanglaite injection	Semen coicis	Anti-HIF^*^		+	Lung cancer	2018	[57]
Yi Ai Fang	From 5 TCM herbs	Anti-HIF^*^		+	CRC	2016	[68]
Pien Tze Huang	Mixture of TCM herbs	↓cancer stem cells	+		CRC	2016	[69]
Curcumin	Curcuma longa	Cancer prevention	+		CRC	2013	[70]
Total glycosides of paeony	Paeoniae radix alba	Autoimmunity control	+		↓adverse effects of ICBs^*^	2020	[48]
Astragalus polysaccharides	Astragalus membranaceus	Enhance Immunity		+	General purpose	2015	[71]
Astragalus polysaccharides	Astragalus membranaceus	Enhance tissue repair		+	General purpose	2018	[72]

* The mode “anti-VEGF” is actually closely related to “anti-HIF”, as hypoxia may lead to increased angiogenesis; thus these two categories may overlap; CRC: colorectal cancer; Ref.: reference; “+”: yes; “↓”: reduction of

**Table 3.** Selected effects of TCMs on PD-1/PD-L1 linkage

**Class of TCM studied**	**ICB expression studied**	**Mode of study**	**Combination**	**Cancer**	**Year**	**Ref.**
Qiyusanlong	PD-1/PD-L1	*In vivo*	Nil	Lung	2016	[73]
Ginsenoside Rg3	PD-L1	*In vitro*	ChT	Lung	2017	[74]
Baicalein	PD-L1	*In vitro*	Nil	HCC	2019	[75]
Gegen Qinlian	PD-1	*In vitro*	Nil	CRC	2019	[76]
Bu-zhong-yi-qi	PD-1/PD-L1	Clinical + *in vitro*	ChT	Gastric	2020	[77]
Ginsenoside Rk1	PD-L1	*In vitro* + *in vivo*	Nil	Lung	2020	[78]
Biochanin A	PD-L1	*In vitro* + *in vivo*	Nil	CRC	2022	[79]
Pien Tze Huang	PD-L1	*In vitro* + *in vivo*	Nil	CRC	2022	[80]

Recently, various classes of TCM preparations have increasingly been found to have suppression of ICB effects. Here, selected effects of TCM on PD-1/PD-L1 linkage are highlighted. Ref.: reference

### The “back to the future” potential

Unexpectedly, in mainland China, Bufalin (a proprietary TCM formula) was based on a successful TCM drug that has a history of more than 1,000 years. Bufalin is a popular multi-target TCM oncology formula extracted from chansu. Interestingly, Bufalin’s actions include (but not limited to) those on HIF and VEGF. Admittedly, chansu has been used for treating cancers in mainland China for many centuries [81]. Currently, Bufalin is often prescribed for HCC, a notoriously aggressive cancer. On HCC, Ling’s group has also reported a comparative study for advanced HCC by either a TCM herbal Jiedu Granule or sorafenib [82]. Although this was not a randomized, controlled study, it was remarkable for TCM to have accomplished, for advanced HCC, a 1-year survival rate of 31.2% against 35.5% (sorafenib). This compares well with China’s usual 1-year survival rate of 35.4% (11 studies) [83]. Another remarkable achievement of TCM is that for postoperative cancer patients, Liu et al. [84] had highlighted a few series of these cases in which TCM had been found to be effective for preventing cancer recurrence and metastases; even the disease-free progression was also prolonged significantly. This shows that TCM may well be a safe and effective oncology modality of treatment, even though robust clinical trials are required for further confirmation.

Accepting the vastly different strategies adopted by Western and TCM, and looking at doxorubicin ChT being widely used for treating HCC (since the 1970s) [85] but had very low response rates (10%). In contrast, TCM, e.g., Jiedu Grannules that contain the herbal ingredient Cremastra appendiculata [86] and homoisoflavanone isolated from it, was recently found to have antiangiogenic effects [87]. Although both doxorubicin and sorafenib are Western medicine, upon comparing doxorubicin and Jiedu Grannules, one might realize that even before sorafenib has been developed, TCM already had similar effects of antiangiogenic actions that is clearly better than monotherapy doxorubicin in the 1970s. Another monotherapy that has unfortunately not been successful is the indoleamine 2,3-dioxygenase (IDO) enzyme [88]. It appears that even if one uses monotherapy to target at a single mechanism of the TME, the success rates may well be unsatisfactory. Although the vasculature may be normalized [23] by either Western or TCM, TCM’s multi-herb, multi-target approach appears to be more durable as it may well tackle significantly more escape mechanisms of the TME. Perhaps we should now have a change in the paradigm of concentrating on mostly monotherapies when we face such a highly evasive TME. Taken together, with TCM’s time-honored efficacy, good safety profile, and eminent affordability, it might be a good alternative candidate for combining effectively with ICBs. A probable “bonus” advantage is that some TCMs would also have the potential of tackling the irAEs of ICBs [47–50].

### To AI’s significant contribution TCM validation

As TCM is known to be highly complex and has imprecise descriptive terms, appropriately, the future of TCM development should go by artificial intelligence (AI) technologies. Feasible ways of achieving this objective are through various network pharmacology techniques, as hitherto unknown mechanisms of TCM may thus be further elucidated despite the apparently undue complexity and profound mystery of TCM. A total of 32 TCM studies had been reviewed with AI modes like data mining (DM) and machine learning (ML), being subdomains of AI. ML can achieve pattern recognition [89]. DM would selectively extract useful information from massive data to discover new and useful patterns; it would also look for meaningful and useful data, e.g., specifically for the relationship between TCM and TME. ML involves discovering algorithms that have the design, study data, and developmental prospects of useful algorithms enabling ML (on its own) while under human directives. Remarkably, studies could be conducted to match and learn (e.g., through blood tests) to correlate with TCM’s pulse palpation and tongue observation parameters for syndrome differentiation [90] (in line with TCM practice). Importantly, for TCM, AI technology may provide more precise answers despite imprecise TCM description terms and the largely anecdotal and even empirical TCM approaches. For instance, AI technologies may help to prescribe the most effective herbal medicines despite herbal medications’ highly complex herbal combinations, let alone countless compounds involved. Nevertheless, robust and well-designed randomized controlled clinical trials are mandatory for confirming TCM practice through enhanced AI technologies.

## Discussion

As the VEGF concept has already been rather well validated both by numerous studies and backed by ample clinical experience including the anecdotal clinical experience of normalization of vasculature, especially cerebral edema. This serves to illustrate the tumor’s makeshift vasculature leading to hypoxia of the TME, as well as significant acidity [91]. As leaky blood vessels may well preclude adequate perfusion, existing hypoxia would thus be aggravated; moreover, as hypoxia is also conducive to angiogenesis, a vicious cycle is thus formed [92] and high interstitial fluid pressure and edema eventually ensue (Table 1). Acidosis has long been known to be a pivotal chemical player of the TME, giving cancer cells an important competitive edge over normal cells, through altering the chemical microenvironment to the advantage of cancer cells. Unfortunately, although various novel strategies have been painstakingly designed to combat hypoxia factors in order to enhance immunity, most probably, monotherapies targeting mainly single mechanisms, e.g., the IDO enzyme, may not work well when the TME has so many “plan Bs” and even “plan Cs” [88]. Thus, despite various initial data on the efficacy of IDO1 inhibitors in cancer immunotherapy, during recent clinical trials, adding epacadostat (an IDO1 inhibitor) to pembrolizumab (an ICB) had not made significant contributions to controlling metastatic melanomas. Unfortunately, as many as 13 clinical trials of IDO1 (combined with ICBs) have thus been suspended, canceled, or downsized.

As various factors, e.g., the HIF within the hypoxic TME are attractive targets for cancer control, during the last two decades, considerable efforts have been undertaken on investigating various HIF inhibitors as potential pharmacological agents. However, these newly developed agents for specific HIF control have yet to be approved, let alone some controversies still exist, e.g., the exact target for acriflavine, a repurposed but ironically potent agent against hypoxia [4, 93, 94]. Understandably, with the TME’s tremendous complexity and undue evasiveness, monotherapies targeting merely on a single specific mechanism may well be less appropriate. With TME’s multiple escaping pathways, probably, this is where the alternative TCM’s multi-agent, multi-targeting approach may be preferred. For instance, Pien Tze Huang, a TCM oncology proprietary preparation, has recently been found to act on HIF, even though it has already been in use for nearly 500 years [66], let alone its remarkable safety profile. Clearly, further research on such a line is warranted. Intriguingly, for oncology treatments, mainland Chinese may even rate TCM as a better alternative to Western medicine. This may be attributable to the apparent success of TCM’s “taming” the “wild” TME so that, ironically, even TCM’s much milder and clearly less toxic agents than ChT could be effective, all because the TME is now overcome [95]. Especially, AI technology has most recently managed to elucidate that TCM is actually scientifically valid despite the considerable vagueness and undue complexity of TCM practice [96]. Taken together, as TCM appears to overcome the TME better than Western medicine, it pays to investigate if combining ICBs with TCM would have a better safety profile even if the effectiveness is similar to existing Western medicine ones [97].

Given the vastly different practice patterns between Western and TCM streams, integrating them is an arduous task. Initially, the essential prerequisites are mutual understanding, acceptance of the vast differences, and sincere mutual respect. A common pivotal condition is having a similar conviction to defeat the obviously extremely evasive TME, for the sake of those very desperate cancer patients. Unfortunately, these patients are desperate as they have already exhausted all approved drugs deemed appropriate for their cancers. Now, with a much better understanding of this extremely evasive TME, a realistic alternative to approved drugs has to be considered, especially if these alternatives also have a good safety profile. Admittedly, prescribing exceptional alternative drugs has to be undertaken by expert hands even though these are on compassionate grounds [98]. Unfortunately, as robust clinical trials leading to approvals usually take several years to complete, meanwhile, one can only resort to off-label drugs. Realistically, to attempt the integration of Western and TCM streams, respective expert teams from the two should work closely together. As for clinical trials, as alluded to in this article, it may be prudent to consider an off-label low-dose bevacizumab (7.5 mg/kg) especially as low-dose bevacizumab is not uncommonly prescribed for cancer patients [99]. Ironically, low-dose bevacizumab is more appropriate because with the full-dose (15 mg/kg), the abrupt back pressure that may be induced in case it so happens that there is a rapid antiangiogenesis effect being rendered and this could predispose to bleeding [100]. Moreover, a meta-analysis has also found the risk of high-grade bleeding to be more frequent with high-dose angiogenesis inhibitors, (including bevacizumab) than those with low-doses [101]. Thus, for HCC (a very aggressive cancer) low-dose bevacizumab may be combined with atezolizumab (as in the IMbrave150 Trial [35] except low-dose bevacizumab is used to reduce the high bleeding rate). Most importantly, combined with a TCM proprietary preparation Kanglaite should be considered actively. Kanglaite has been approved by the SFDA ever since 1997 and has since been in extensive clinical use in mainland China [54]. Especially, as Kanglaite has also been found to inhibit HIF-1α [57], its combination with atezolizumab (an ICB) and low-dose bevacizumab (for antiangiogenesis) may widen the scope of activities or have mutual enhancements. Admittedly, Kanglaite has to be duly prescribed by experienced TCM practitioners preferably in a hospital setting. Incidentally, this injectable TCM drug may help to bypass the usual TCM practice of syndrome differentiation (varying prescriptions according to day-to-day clinical variations) [90]. Likewise, batch variations of raw herbs have to be overcome [59]. Although injectable TCMs may help to solve these two problems, clearly, syndrome differentiation is still hailed by most TCM practitioners, especially those more experienced ones. This very basic difference between the two medical streams could be the worst stumbling block to integrating the two.

## Conclusions

With the TME as the worst culprit causing undue cancer resistance, much effort has already been spent on developing specific targets against the TME, e.g., during the past two decades, considerable efforts targeting HIFs have already been undertaken except all these newly developed agents have yet to be approved [4]. In contrast, even in the last century, China’s SFDA has already approved Kanglaite which may be an anti-HIF agent [57]. Moreover, apart from anti-HIF agents, TCM also has various herbs that have antiangiogenic or anti-VEGF properties. Although much more research work is called for, for the highly evasive TME, as TCM’s multi-agent, multi-target approach seems more advantageous, it might be the time for a paradigm change by adopting the multi-target approach against the TME. This appears to be the best chance to enhance the low response rates of ICBs without adding significant toxicity. Importantly, TCM’s clinical trials are usually not robust enough, given that TCM has not only problems with herbal batch variations but also the well-established clinical practice of “syndrome differentiation” at each clinical visit may prelude strictly uniform prescriptions for clinical trial purposes. Clearly, these two points must be overcome. Although the road to overcoming cancer resistance is still a long one, there seems to be light at the end of the tunnel.

## Abbreviations

AI: artificial intelligence
ChT: chemotherapy
CTLA-4: cytotoxic T-lymphocyte-associated-4
DMARDs: disease-modifying anti-rheumatic drugs
FIH: factor inhibiting hypoxia-inducible factor
FMT: fecal microbiota transplantation
HCC: hepatocellular cancer
HIF: hypoxia-inducible factor
ICBs: immune checkpoint blockers
IDO: indoleamine 2,3-dioxygenase
irAEs: immune-related adverse events
linacs: linear accelerators
mChT: metronomic chemotherapy
ML: machine learning
NSCLCs: non-small cell lung cancers
ORR: objective response rate
PD-1: programmed death-1
PD-L1: programmed death-ligand 1
RCC: renal cell carcinoma
SFDA: State Food and Drug Administration
TCM: traditional Chinese medicine
TILs: tumor-infiltrating lymphocytes
TKIs: tyrosine kinase inhibitors
TME: tumor microenvironment
Tregs: regulatory T cells
VEGFs: vascular endothelial growth factors

## Declarations

### Copyright

© The Author(s) 2022.
